# School for the Indigent Blind

**Published:** 1901-11-23

**Authors:** 


### SCHOOL FOR THE INDIGENT BLIND.

### LAYING THE FOUNDATION STONE.

On Wednesday, 13th inst., H.R.H. Princess Christian of Schleswig Holstein laid the foundation stone of a new school at Leatherhead for the indigent blind, the purchase of the old schools at Southwark by the Baker Street and Waterloo Railway Company having made it necessary to remove the pupils to the country. The town of Leatherhead was gaily festooned with flags and mottoes for the occasion, and a very large number of friends and supporters of the institution travelled from Waterloo by special train. The ceremony, which was over in half an hour, began with the National Anthem, led by the band of the Royal Engineers, and the presentation of bouquets to the three Princesses (Princess Christian being accompanied by her daughter and niece) by the children of the principal, the Rev. St. Clare Hill.

A brief address was next read by Major-General J. E. D. Hill, vice-president and chairman of the building committee. In it he said that the school in Southwark was opened in the first year of the reign of Queen Victoria, though the work for the blind was begun in 1799, a hundred and one years ago. Since that time more than 3,500 blind persons had received the benefits of the institution. When the Railway Bill authorising the purchase of the site of the schools in St. George's Circus was presented to Parliament, the committee felt that they could not with justice or reason oppose it; they considered also that the health of the pupils would be greatly benefited by removal into the country. No pains had been spared to make the new buildings thoroughly efficient and replete with every modern requirement; and to this end the principal and the architect had visited many schools in England, on the Continent, and in America, before the plans were drawn out. There would be accommodation for 200 pupils, and every care had been taken to provide for their physical and mental well being; for example, the workshops would be used for no other purpose; there would be cloisters where exercise could be taken in bad weather, and there would be a gymnasium with every kind of appliance. The committee looked forward with pride to the completion of the buildings, and they hoped that through their efforts a school would be given to the nation which would be in every way worthy of the objects for which it was founded. Though the buildings were designed to accommodate 200 pupils only, he saw no reason why they should not be increased so as to take in 300 or even more. The stability of the work was regarded by the committee as of primary importance, and in all their efforts they would keep in mind the motto of the school—"to render the blind self-reliant by teaching them a trade."

After the singing by the Blind Choir of a "Song of Welcome," words by pupil H. Blake and music by pupil S. Brooks, and a hymn, also composed by H. Blake, prayers were said by the Lord Bishop of Winchester, after which the stone was laid. The purses presented to the Princess for the fund included one containing £300 from the Chairman, and £10 from the Merchant Taylors' Company.

The new Lord Mayor of London (Sir Joseph Dimsdale), in proposing a resolution of "Prosperity to the School," alluded to the early days of the work in the old Tea Gardens in Southwark, known as the "Dog and Duck." The resolution was seconded by the Vicar of Leatherhead, the Rev. Canon Utterton, who gave the school a very hearty welcome on behalf of the people of Leatherhead; and a vote of thanks to H.R.H. Princess Christian, moved by Alderman Sir Joseph Savory and seconded by Mr. R. K. Causton, M.P. for Southwark, brought the ceremony to a close.

The new buildings are situated to the south-west of the town, on the Highlands Road; the site is irregular in shape, and on sloping ground, which necessarily governs to a large extent the arrangement of the buildings. These consist of class-rooms, workshops, recreation rooms, dining hall, dormitories, etc., for about 100 boys on the one side, with similar accommodation for 100 girls on the other side, with a central hall, offices, kitchen department, etc., common to both the boys and the girls in the middle. On either side of the central hall there is a courtyard surrounded by covered corridors, in which the pupils will be able to take exercise in wet weather. The buildings, which are now nearly ready for roofing in, are of red brick facings with stone dressings, and will be covered with grey green slates; the floors will be of fireproof construction by Messrs. Homan and Rodgers, and there will be ample provision for escape in case of fire by two staircases to each side internally, and iron staircases externally; in addition there will be numerous fire hydrants with a good pressure of water. A large amount of glazed brickwork has been introduced, and the corridors will be paved with marble mosaic. The heating is to be by low-pressure hot water, and is being executed by Messrs. Strode and Co. In addition to the main buildings there will be a chapel, gymnasium, laundry, sanatorium, principal's residence and lodge; while a swimming bath may possibly be added later on.

The architect is Mr. C. Pemberton Leach, of Pelham Crescent, London, and Messrs. Foster and Dicksee, of Rugby, are the contractors for the building.

